# Shelter from the cytokine storm: pitfalls and prospects in the development of SARS-CoV-2 vaccines for an elderly population

**DOI:** 10.1007/s00281-020-00821-0

**Published:** 2020-11-06

**Authors:** Annalisa Ciabattini, Paolo Garagnani, Francesco Santoro, Rino Rappuoli, Claudio Franceschi, Donata Medaglini

**Affiliations:** 1 Laboratory of Molecular Microbiology and Biotechnology (LA.M.M.B.), Department of Medical Biotechnologies, University of Siena, Siena, Italy; 2 Clinical Chemistry, Department of Laboratory Medicine, Karolinska Institute at Huddinge University Hospital, SE-171 77 Stockholm, Sweden; 3 Department of Experimental, Diagnostic and Specialty Medicine (DIMES), University of Bologna, 40139 Bologna, Italy; 4 Interdepartmental Centre ‘L. Galvan’ (CIG), University of Bologna, Via G. Petroni 26, 40139 Bologna, Italy; 5 GSK, Siena, Italy; 6 vAMRes Lab, Toscana Life Sciences, Siena, Italy; 7 Faculty of Medicine, Imperial College, London, UK; 8 Lobachevsky State University, Nizhny Novgorod, Russia

**Keywords:** COVID-19, SARS-CoV-2, Vaccination, Older population, Immunosenescence, Inflammaging

## Abstract

The SARS-CoV-2 pandemic urgently calls for the development of effective preventive tools. COVID-19 hits greatly the elder and more fragile fraction of the population boosting the evergreen issue of the vaccination of older people. The development of a vaccine against SARS-CoV-2 tailored for the elderly population faces the challenge of the poor immune responsiveness of the older population due to immunosenescence, comorbidities, and pharmacological treatments. Moreover, it is likely that the inflammaging phenotype associated with age could both influence vaccination efficacy and exacerbate the risk of COVID-19-related “cytokine storm syndrome” with an overlap between the factors which impact vaccination effectiveness and those that boost virulence and worsen the prognosis of SARS-CoV-2 infection. The complex and still unclear immunopathological mechanisms of SARS-CoV-2 infection, together with the progressive age-related decline of immune responses, and the lack of clear correlates of protection, make the design of vaccination strategies for older people extremely challenging. In the ongoing effort in vaccine development, different SARS-CoV-2 vaccine candidates have been developed, tested in pre-clinical and clinical studies and are undergoing clinical testing, but only a small fraction of these are currently being tested in the older fraction of the population. Recent advances in systems biology integrating clinical, immunologic, and omics data can help to identify stable and robust markers of vaccine response and move towards a better understanding of SARS-CoV-2 vaccine responses in the elderly.

## Older people as the main target population for a COVID-19 vaccine

**Fig. 1:**
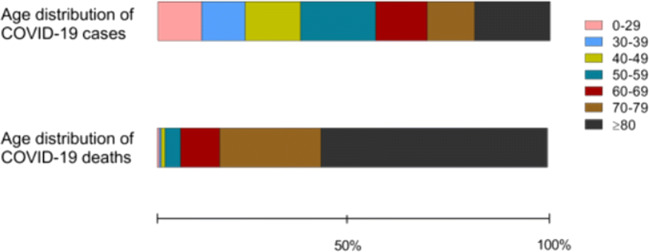
Distribution of COVID-19 cases and deaths by age group. Frequency of COVID-19 cases (upper diagram) and deaths (lower diagram) among different age ranges (colored boxes) in Europe, estimated in July 2020

The present SARS-CoV-2 pandemic is posing an unprecedented healthcare and socio-economic burden worldwide. SARS-CoV-2 hits greatly the older and more fragile fraction of the population, boosting the evergreen issue of vaccination in elderly people. In Europe, as of week 39/2020, SARS-CoV-2 infection was reported in over 5.7 million people; of those, about 45% were aged 60 or more, while more than 90% of the 235,000 reported deaths occurred in this age group. Strikingly, people aged 80 or more accounted for more than 50% of the reported deaths, with a median age at death of 81 years (https://www.euro.who.int/en/health-topics/health-emergencies/coronavirus-covid-19/weekly-surveillance-report; Fig. 1). These figures are in line with estimates elaborated from the epidemiological data collected in China at the beginning of the outbreak, which reported an adjusted case fatality ratio of 9.5% in the ≥ 60 age population [1]. The male to female ratio of SARS-CoV-2 reported cases is around 0.86, while the M:F ratio of deaths is around 1.38, suggesting that, despite being more frequently infected, females are more capable of dealing with the infection [2, 3].

It is widely reported that most deaths occurred among patients with at least one underlying disease, such as hypertension [4] and diabetes mellitus [5]. A meta-analysis of seven clinical studies performed in China identified chronic obstructive pulmonary disease (COPD), cardiovascular disease, and hypertension as risk factors for severe disease and intensity care unit (ICU) admission [6]. Analysis of risk factors associated with more than ten thousand deaths by COVID-19 in the UK confirmed that age was linearly correlated with risk of death and that obesity, diabetes, severe asthma, respiratory disease, neurological disease (including stroke), recent (< 5 years) hematological malignancy, and recent (< 1 year) cancer diagnosis were all associated with higher death risk. As for hypertension, the hazard risk was higher only for the population < 70 years old, even if hypertension itself was strongly associated with other risk factors such as obesity and diabetes [7]. Importantly, these epidemiological studies identify the categories of subjects who are at higher risk of developing severe SARS-CoV-2 infection and that should be prioritized in vaccine administration.

The massive effort for the development of a vaccine against SARS-CoV-2 could be frustrated by the poor responsiveness to vaccination that characterizes a large proportion of the elderly population. In this rush against the time, we risk to pay a dear toll for the lack of knowledge in the response to vaccination of the elderly, a well-known issue, neglected notwithstanding its evident urgency and the annual reproposal through the seasonal influenza epidemic above all. Interestingly, there is a consistent overlap between the factors hampering vaccination effectiveness in the elderly and those that boost the virulence and worsen the prognosis of SARS-CoV-2 infection.

A common characteristic of the elderly people is the onset of a sterile low-grade increase of the basal inflammatory state named “inflammaging,” which is considered a universal etiological agent of most of the age-related diseases [8]. It is likely that some specific components of the inflammaging phenotype could both influence vaccination efficacy and then increase the risk of the early massive production of inflammatory cytokines, termed the “cytokine storm syndrome.” This is a condition reported in severe COVID-19 cases during which the patient’s immune system spins out of control and starts damaging healthy organs owing to the increased vascular permeability, vascular paralysis, and hypovolemic shock [9].

Angiotensin-converting enzyme 2 (ACE2) has been identified as the receptor for SARS-CoV-2, and it has been suggested that differential levels of ACE2 in the cardiac and pulmonary tissues of younger versus older adults may be at least partially responsible for the spectrum of disease virulence observed among patients with COVID-19 [10].

Here, we analyze the different aspects that tackle SARS-CoV-2 vaccination in the elderly population, considering immunologic, genetic, and socio-economic factors that impact on the age-related changes of immune responses. A view of the current available vaccine platforms with a special focus on the clinical trials including older adults is reported.

## How the elderly condition can affect COVID-19 disease progression and the response to vaccination

### Immunosenescence

For many reasons, it is difficult to clearly define what immunosenescence is: (i) immunosenescence is quite complex and involves cellular and molecular changes occurring lifelong (from newborns to centenarians) in both the innate and the adaptive immune systems; (ii) these changes can be at the same time detrimental and beneficial/adaptive [11]; (iii) it is difficult to identify a unique common marker of immunosenescence, due to the overwhelming number of biological and non-biological factors that can impinge lifelong on the immune system of each individual; (iv) the changes occurring with age in the immune system are deeply correlated with the profound environmental, epidemiological, lifestyle, societal, medical, and public health changes, including vaccination policies and practices, that occurred in the last century.

Accordingly, immunosenescence is highly *context-dependent* [12], different in different geographical and historical settings and in men and women, correlated to socio-economic position, and sensitive to psychological stressors. Indeed, both the adaptive and the innate immune systems have the capability of “remembering” all immunological stimuli a person has been exposed to lifelong. We have conceptualized this situation with the term *immunobiography*, which should help in understanding the enormous heterogeneity of the immune phenotype in old people. This is also the reason why there is a sort of imprinting in the immune responses favoring those towards antigens that have been experienced early in life [13].

The complex biological processes of aging are the result of alterations in gene regulation and protein expression, signaling pathways, and biological networks. Complex changes, including pervasive epigenetic and metabolic modifications, affect most of the subsets of naïve, memory, regulatory effector T cells, and B cells [14–16]. Despite the challenging complexity, a universally observed hallmark of immunosenescence is the decrease of naive T cells (particularly CD8+ T cells) in peripheral blood [17] consequent to thymic involution responsible for the early decline in the output of naïve T cells to the periphery and for the related shrinking of the T cell repertoire [18–20]. Other important aging-related alterations are (i) the shift in the bone marrow maturation of hematopoietic cells towards myelocytic differentiation [21], concomitant with a reduced lymphopoiesis, mainly due to changes in progenitor cells in the bone marrow [12, 22]; (ii) the increased numbers of memory cells owing to large clonal expansion towards epitopes of persistent viral infections (Cytomegalovirus [CMV] and Epstein Barr virus [EBV]) [23, 24]; (iii) the compromised ability of CD4+ T cells to differentiate into functional subsets, resulting in a multitude of dysregulated responses, such as a reduced cognate help to B cells with consequent reduced humoral immunity, and the increased ratio of the proinflammatory Th17 cells with respect to the immunosuppressive T regulatory cells, thus favoring a basal proinflammatory status [16, 25]; (iv) accumulation of differentiated exhausted T cells, induced by a repeated pathogen encounter during chronological aging, and end-stage differentiated senescent T cells, characterized by a progressive reduction of telomere length leading to a state of proliferative arrest [26].

With aging, health conditions associated with immune senescence, comorbidities (particularly noncommunicable diseases such as heart disease, cancers, and metabolic and autoimmune diseases), and pharmacological treatments affect the immune responses to both vaccines and infectious diseases.

Overall, as a result of immunosenescence, the elderly population is more susceptible to infections, particularly to influenza, *Streptococcus pneumoniae* RSV, and group B streptococcus but also to opportunistic, re-emergent chronic infections such as herpes zoster as well as antibiotic-resistant nosocomial pathogens.

The reduced adaptive immune response, together with altered innate cell function, such as chemotaxis, phagocytosis, signaling pathways, and intracellular killing, prevents the appropriate control of the initial inflammatory response elicited upon viral infection. For RNA virus, such as coronavirus, different pattern recognition receptors (PRR) are triggered on the innate cells during the early phases of infection. These include the endosomic Toll-like receptor 3 and 7 and the cytosolic RIG-I/MDA-5 molecules, which recognize viral RNA [27], and the cGAS-STING pathway, which recognizes cytosolic DNA [28] activated by cellular damage and mitochondrial DNA release caused by viral infection [29]. The stimulation of these PRR leads to the expression of type I IFN, a factor that limits viral replication through the stimulation of interferon-stimulated genes, and other inflammatory cytokines [30]. For Middle East respiratory syndrome (MERS)-CoV, the timing of type I IFN production appears to dictate the outcome of infection in mouse models, and its administration within 1 day after infection was protective against lethal infection, while a delay in IFN production caused an inability to control viral replication, leading to cellular damage of airway epithelia and the lung parenchyma and an eventual lethal inflammatory cytokine storm [31]. The latter response often predominates in older individuals and in aged mouse models of SARS-CoV-1 infection [32, 33].

**Fig. 2:**
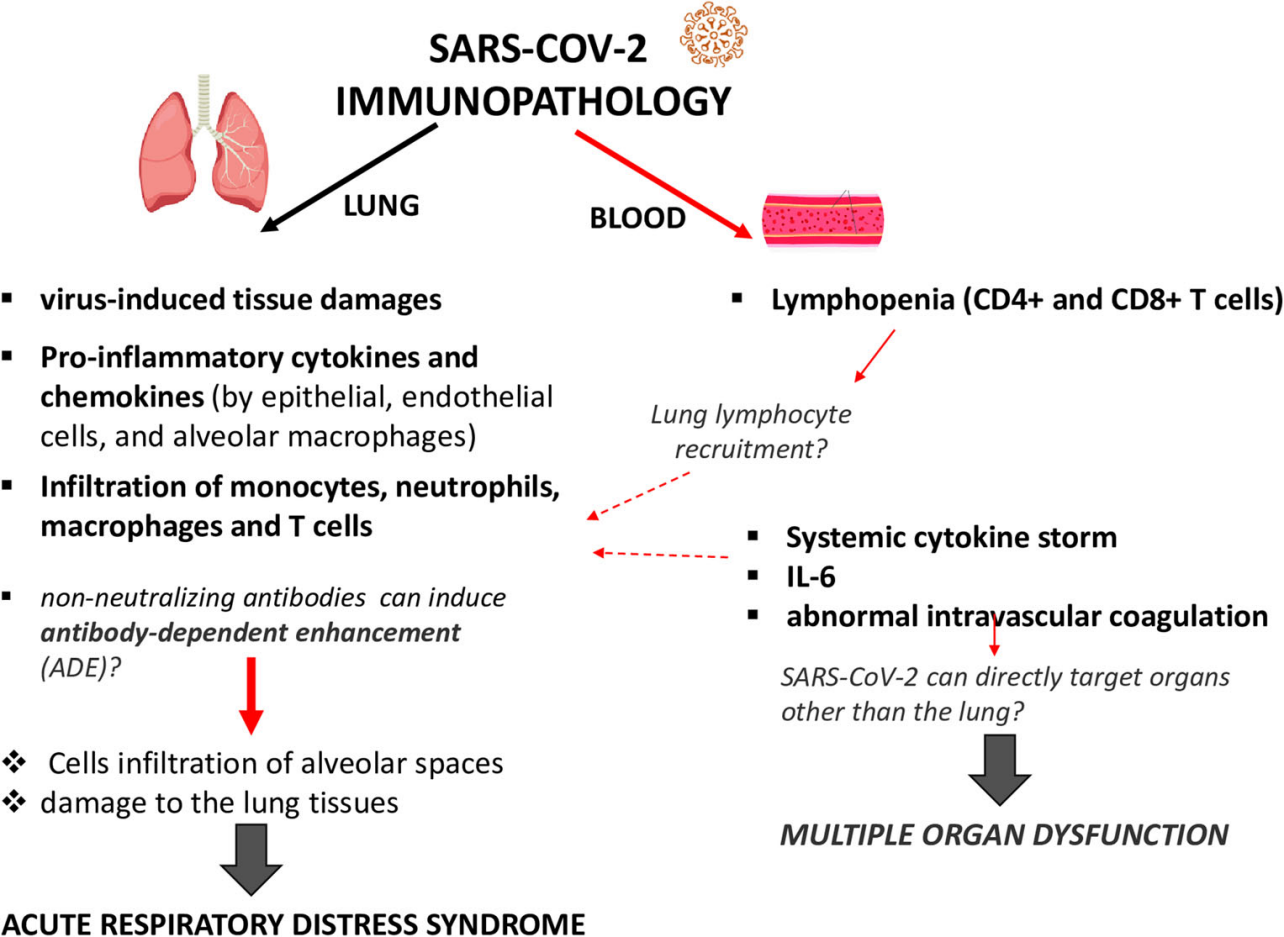
Possible mechanisms of SARS-CoV-2 immunopathology. Systemic and local (lung) immune responses and their pathological role, following SARS-CoV-2 entry into the host are schematically represented. Induction of innate immune responses is a crucial step in the pathophysiology of COVID-19 disease, contributing to hyperinflammation and tissue damage during the later stages of the disease. Infiltration of immune cells in the lungs causes overproduction of proinflammatory cytokines, which eventually damages the lung infrastructure, accumulation of macrophages in the air spaces and diffuse alveolar damage leading to acute respiratory distress syndrome (ARDS). Furthermore, elevated levels of circulating proinflammatory cytokines can cause septic shock and multi-organ dysfunction. Together with the hyperinflammatory response, overt disseminated intravascular coagulation has been reported and a significant lymphopenia, mainly related to CD4+ T and CD8+ T cells, has been observed, possibly due to pulmonary recruitment of lymphocytes from the blood. A possible immunopathological role can be mediated by non-neutralizing antibodies produced by B cells, which may enhance SARS-CoV-2 infection through antibody-dependent enhancement (ADE), further exacerbating organ damage

Induction of innate immune responses is a crucial step in the pathophysiology of COVID-19 disease (Fig. 2). On one hand, it triggers the anti-viral host defense mechanisms necessary for elimination of infection, but on the other hand, it may contribute to hyperinflammation and tissue damage during the later stages of the disease in a minority of patients [34]. This can be particularly relevant in the elderly population in which inflammaging, the state of chronic low-grade sterile inflammation [8], characterized by high serum concentrations of C-reactive protein (CRP), IL-6, IL-8, and tumor necrosis factor (TNF)-α, can be present.

### Inflammaging

Tissue damage in COVID-19 is mainly mediated by an excess of immune response to the virus, which results in a cytokine storm, with activation of the IL-6 signaling pathway. The pathophysiology of SARS-CoV-2 infection has strong similarities to other severe viral lung infections caused by SARS-CoV-1 and MERS-CoV.

One of the first published studies on clinical features of COVID patients hospitalized in Wuhan showed that proinflammatory cytokines and chemokines, such as TNF-α, granulocyte-colony stimulating factor (G-CSF), interferon gamma-induced protein-10 (IP-10), monocyte chemoattractant protein-1 (MCP-1), and macrophage inflammatory proteins 1-α (MIP-1α), were significantly higher in patients admitted to the intensive care unit (ICU) compared to those who were not in ICU [35]. Immune pathology in the form of vascular and cutaneous lesions has also been widely reported [36, 37]. The role of a dysregulated inflammatory response was proven in an animal model of SARS-CoV-1 infection using aged macaques. Aged animals are more prone to develop severe disease and activate more readily the innate response, in particular the NF-kB pathway and proinflammatory cytokines such as IL-8 and IL-1β, while not inducing significantly IFN-β response. The innate immunity activation is not due to the viral load, which is comparable among young and aged macaques [38].

Transcriptomic analysis performed in samples from subjects with severe COVID-19 revealed the presence of low levels of type I and type III interferon genes together with elevated levels of proinflammatory cytokines and chemokines, such as IL-6, IL1RA, CCL2, CCL8 CXCL2, CXCL8, CXCL9, and CXCL16 [39].

Which type of cells elicits this cytokine storm and the virological mechanisms behind this inflammatory reaction are still unclear [40]. Lung epithelial cells, alveolar macrophages, dendritic cells, and endothelial cells can effectively release the proinflammatory cytokines and chemokines, thus attracting monocytes, macrophages, and T cells to the site of infection [41]. The overproduction of proinflammatory cytokines in the lungs can damage the tissue infrastructure, recruit macrophages that infiltrate air spaces, and generate the respiratory failure from acute respiratory distress syndrome (ARDS), which is recognized as the leading cause of mortality. Meanwhile, the direct attack on other organs by disseminated SARS-CoV-2, the immune pathogenesis caused by the systemic cytokine storm, and the microcirculation dysfunctions together may lead to multi-organ damage, even though whether SARS-CoV-2 can directly target organs other than the lung and how it can happen are aspects that need to be further investigated [40] (Fig. 2).

Together with the hyperinflammatory response, a significant lymphopenia, mainly related to CD4+ T and CD8+ T cells, which correlates with the severity of viral infection, was reported [42–44]. The causes of this adaptive immunity suppression are still unclear. Pulmonary recruitment of immune cells from the blood and the infiltration of lymphocytes into the airways may explain the reduction in blood. The well-known age-related alteration of the immune function of T cell and B cells could lead to insufficient control of viral replication, thus increasing the macrophage infiltration and the lung injury (Fig. 2).

Finally, a possible immunopathological role can be mediated by non-neutralizing antibodies produced by B cells that may enhance SARS-CoV-2 infection through antibody-dependent enhancement (ADE), further exacerbating organ damage. It has recently been shown that SARS-CoV-1 and the MERS-CoV take advantage of non- or subneutralizing antibodies and enter cells via surface CD32a receptors, an Fc receptor expressed on the surfaces of monocytes and alveolar macrophages. The antibody-CD32 interaction facilitates viral entry and infection, and activates intracellular signaling to upregulate proinflammatory cytokines [45].

**Fig. 3:**
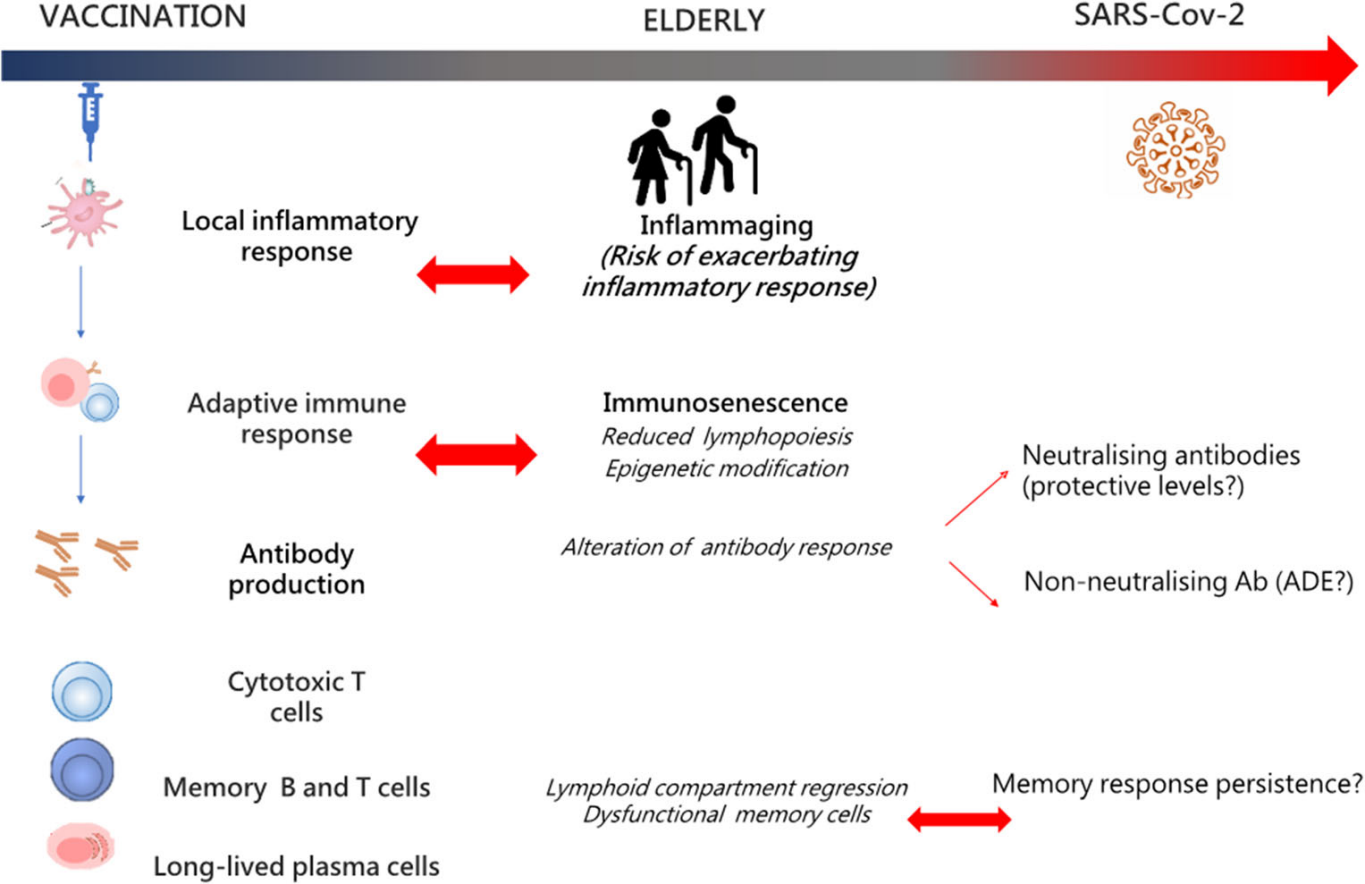
Challenges for the development of a SARS-CoV-2 vaccine for elderly people. Schematic interconnection between the main immune mechanisms elicited by the vaccination process, with the peculiarity of the elderly immune system—affected by both inflammaging and immunosenescence—and the still undefined correlates of protection from SARS-CoV-2 infection. The complex and still unclear immunopathological mechanisms of SARS-CoV-2 infection, together with the progressive age-related decline of innate and adaptive immune responses, and the lack of a clear correlate of protection make the design of vaccination strategies for older people extremely challenging

The complex and still unclear immunopathological mechanisms of SARS-CoV-2 infection, together with the progressive age-related decline of innate and adaptive immune responses, and the lack of a clear correlate of protection, make the design of vaccination strategies for older people extremely challenging (Fig. 3).

### Biological age

An emerging class of instruments in the aging research is the development of markers capable of assessing the speed of the aging process. Age is a major risk factor for a high number of diseases, and in general, it affects the fitness of each individual, including the capability of responding to vaccine administration and counteracting a severe infection [46]. However, it is also evident that the elderly population is extremely heterogeneous, so while chronological age is useful to identify macroscopic risk classes, it is poorly informative within age classes to get individual information. Biological age is thus useful to evaluate clinical parameters and health risks on the basis of the individual aging pace, which tend to be more heterogeneous in the elderly population. Several established biological age markers have been generated based on both classical anthropometric, clinical, and biochemical parameters as well as on innovative molecular characterizations such as DNA methylation and the composition of the N-glycan shell of circulating proteins [47]. Such biomarkers have shown in a number of studies that the aging pace is higher in the vast majority of the different elderly conditions, thus demonstrating that biological age assessment should be a critical information in a broad spectrum of clinical practices and in the development of strategies to tackle healthcare burden and emergencies. The detailed description of available biological age markers is out of the scope of the present manuscript, and an extensive overview is available in the review by Jylhävä et al. [46]. To date, biological age has not been assessed in the SARS-CoV-2 clinical setting, but it is noteworthy that biological age has been associated with all the most important risk factors related to a poor prognosis of SARS-CoV-2 infection. The field of elderly vaccination could benefit from biological age information, but also in this case, the available data are rare. In a study from Gensous et al. [48], the whole genome methylation profile of PBMC was assessed in a group of volunteers of different ages who underwent influenza vaccination. The relationship between the vaccination response and the methylation profile was studied. While no difference in terms of biological age emerged in the study, an age-dependent epigenetic remodeling emerged in elder non-responders. The study is limited owing to the very low number of analyzed subjects but confirmed that DNA methylation is an informative instrument to be exploited in vaccination studies and strategies.

### Immunobiography

Immunobiography refers to the comprehensive immunological, clinical, socio-economic, and geographical history of each individual, and accounts for the large heterogeneity observed in the elderly regarding their health status, mirrored by their large individual variation in the responsiveness to vaccines. A major advantage of immunobiography is that it incorporates the most advanced conceptualization of *immunosenescence* which, according to the most recent literature [49], has to be considered as a context- and population-dependent phenomenon. Accordingly, in order to be properly interpreted, age-related changes of immune parameters occurring in an elderly person necessitate a variety of other additional data regarding sex/gender, demographic cohort, population/country, individual immunological history, anthropometric parameters, socio-economic status and education, CMV serostatus, morbidity and co-morbidity, among others. It is of critical importance taking in consideration the elderly vulnerability to direct the rational design of vaccines designed for this target population.

### Gender

Gender is a critical issue in both vaccination of the elderly and in the SARS-Cov-2 pandemic. The pandemic epidemiological data show clearly that the risk of severe disease and mortality is sharply higher in men than in women. Men’s hospitalization exceeds women by about 50%, indicating a significantly higher susceptibility of men towards severe SARS-CoV-2 infection. Available data show that men outnumber women 2 to 4 times in terms of ICU admissions [50–52]. These numbers are concordant with the fatality rate that ranges between 1.2 and 1.4 men deaths for one women death. Moreover, this unbalanced pattern is mirrored by the vaccine uptake, responses, and outcome in older-aged individuals. Elderly women are indeed more responsive than men for several vaccine protocols recommended in older-aged individuals such as those against influenza, tetanus, pertussis, shingles, and pneumococcal infections [53]. On the other hand, an influenza vaccination study reported that aged men antibodies had higher affinity than those produced by women. Moreover, men seem to respond better to pneumococcal vaccination in two independent studies [54, 55]. There is an impaired vaccination response in both old men and women with sex-specific weaknesses. The most striking data, however, is related to infection and all-cause mortality: indeed, in a number of reports, vaccine administration produces a sharper decrease of specific and all-cause mortality in vaccinated women compared to men, indicating that women have higher benefit from vaccination in the elderly [56–58]. These data indicate the need to consider sex-specific vaccination protocols for the elderly population [58, 59] and that the lack of such instruments could be critical in the SARS-CoV-2 pandemic since old men are both the most susceptible to severe SARS-CoV-2 infection and are those less likely protected by a possible SARS-CoV-2 vaccine [60–62].

### Microbiota

Another factor that could affect vaccine response is the intestinal microbiota that plays a crucial rule in the regulation of the immune system and is highly affected by age [63–66]. Microbial community composition indeed is influenced by age, environmental and socio-economic factors, diet, gender, chronic infections, immunosuppressive chemotherapy, antibiotic treatment, or probiotic use [64, 67–69]. The improvement in the nucleic acid sequencing obtained in the last 15 years hits massively the microbiological research and promotes the analysis of heterogeneous microbiological ecosystems such as those that reside in humans. The characterization of such ecological niches opens to the new conceptualization of humans as metaorganisms (organisms composed of different organisms) to stress the tight interdependencies between the host and the microbiological species residing in different anatomical districts.

Gut microbiota changes with age and that is likely an important contributor and modulator of the inflammaging phenotype [70, 71]. Elderly people have less diverse gut microbiota and reduced beneficial microorganisms [72]. The general imbalance of gut microbiota, called “dysbiosis,” is associated with both frailty, a geriatric syndrome leading to increased vulnerability for adverse health outcomes, and systemic inflammation. Since a hyperinflammation status has been observed in most severe cases of SARS-CoV-2 infection, it is possible that gut dysbiosis may influence the clinical manifestation in COVID-19 infection [73, 74].

Interestingly, the gut microbiota has been shown to also affect pulmonary health through a bidirectional cross-talk between the gut microbiota and the lungs [75]. Along this “gut-lung axis,” microbial products can reach the lung through blood and modulate pulmonary immune responses [76], while inflammation processes occurring in the lung can impact on the gut microbiota [77]. Some studies have demonstrated that respiratory infections are associated with a change in the composition of the gut microbiota [78] and the antibiotic treatment of mice for removing some gut bacteria has led to increased susceptibility to influenza virus infection in the lungs [79]. Since one of the severe clinical manifestations of COVID-19 is pneumonia and progression to acute respiratory distress syndrome (ARDS), especially in elderly and immune-compromised patients [80], it can be speculated that SARS-Cov-2 infection can affect this gut-lung cross-talk which might influence the outcome of the clinical manifestation [81]. Moreover, even though respiratory symptoms represent the principal clinical presentation of COVID-19, clinical evidence suggests that the intestine may be another viral target organ. Indeed, a high expression of ACE2 has been observed in the brush border of intestinal enterocytes [82] and, using a human small intestinal organoid system, it has been demonstrated that SARS-CoV-2 readily replicates into the enterocytes, resulting in the production of large amounts of infective virus particles [83]. Some reports show that SARS-CoV-2 RNA can be detected in the stool of some patients of COVID-19 [84, 85], and patients often present gastrointestinal symptoms such as diarrhea, vomiting, and abdominal pain [86]. Therefore, the characterization of the gut microbiota in patients with active SARS-CoV-2 intestinal infection could represent a striking aspect to investigate.

These considerations on inflammaging, immunobiography, biological age, gender, and microbiota pertain to every vaccination strategy, but are particularly relevant for the development of vaccines against SARS-CoV-2 since it more seriously affects the elderly population and immunopathology is a crucial factor for the severity disease.

## Need for the design of a SARS-CoV-2 vaccination strategies tailored for the elderly

SARS-CoV-2 vaccines are urgently needed, and their design should take into consideration that the elderly population is the main target population for vaccination. While older adults are most likely to be severely affected by COVID-19, they also may be less responsive to vaccination. Efficacy of vaccination in the elderly is indeed strongly reduced compared to that of younger adults [87, 88]. SARS-CoV-2 vaccination strategies, tailored for the elderly, should take into consideration the delicate balance between immunosenescence/inflammaging and the immunopathological aspects of the COVID-19 disease (Fig. 3). Vaccine adjuvants and vectors should be specifically designed for stimulating the elderly immune system without exacerbating the inflammatory status [87]. Despite these considerations, the elderly are rarely included in vaccine clinical trials; in the last decades, the vast majority of randomized control trials did not include older adults and in particular frail older adults who are mostly at risk. We currently do not have full knowledge on the mechanisms of immunity to protect this population from SARS-CoV-2 [10].

The development of a SARS-CoV-2 vaccine is extremely challenging, since we are faced with a novel virus, just emerged in humans, and correlates of protection have not yet been fully identified, even though the induction of neutralizing antibodies is presumed to be a crucial target for an effective vaccination (Fig. 3).

Protection in older individuals against influenza virus appears to require higher neutralization titers than in younger individuals [89], and this issue might need to be addressed for SARS-CoV-2. The knowledge obtained from the vaccine development efforts for MERS and SARS-CoV-1 can be of high value for SARS-CoV-2, although no vaccines are licensed for these coronavirus strains [90].

**Fig. 4:**
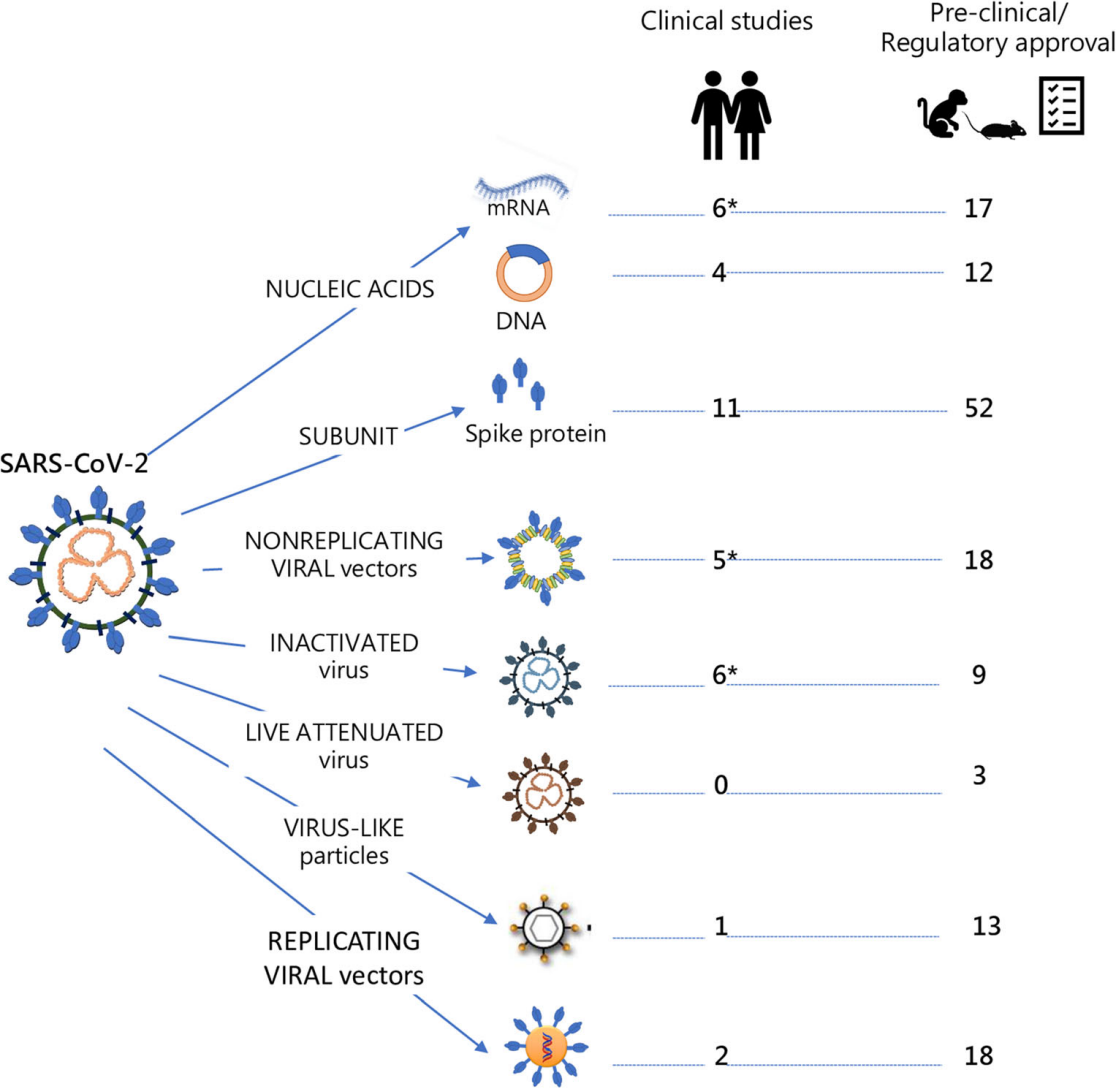
SARS-CoV-2 vaccine candidates based on different vaccine platforms. Schematic representation of the different vaccine platforms used for developing SARS-CoV-2 vaccines. These include nucleic acid (both mRNA and DNA); subunit S protein with different adjuvants; non-replicating viral vectors (such as Adenovirus); inactivated SARS-CoV-2 virus alone or combined with adjuvants; live SARS-CoV-2 attenuated virus; virus-like particles and replicating viral vectors (such as Measles virus, Influenza virus, Vesicular stomatitis virus, and others). About 140 vaccine candidates are currently involved in pre-clinical studies, while 35 vaccine candidates are worldwide tested in clinical studies, and some of them (indicated with *) have already reached the phase III. For each platform, the number of ongoing clinical or pre-clinical studies is reported. Data are referred to the WHO report, updated to 17 September 2020

Memory CD4+ T cells, induced by infections with other coronavirus and capable of responding to SARS-CoV-2, have been detected in 20–50% of SARS-CoV-2 unexposed donors [91, 92]. The characterization of these cross-reactive T cells in the elderly and their impact on the immunogenicity of vaccine candidates should be taken into consideration in the ongoing COVID-19 vaccination studies. SARS-CoV-2 vaccine candidates based on different vaccine platforms have been developed, and about 140 candidates have been tested in pre-clinical experiments, according to the WHO landscape documents of COVID-19 candidate vaccines (https://www.who.int/publications/m/item/draft-landscape-of-covid-19-candidate-vaccines) (Fig. 4). Information on the specific SARS-CoV-2 molecules selected as vaccine antigens is limited, even though most candidates aim to elicit neutralizing antibodies against the spike (S) protein and its receptor-binding domain (RBD), as already performed with the SARS and MERS vaccines. A wide range of both innovative and traditional technology platforms has been deployed, including nucleic acid (DNA and RNA), recombinant viral vectors (replicating and non-replicating), recombinant protein combined with adjuvants, and live attenuated or inactivated virus [93]. Some of these platforms were already tested in human studies for SARS-CoV-1 virus, such as inactivated virus, DNA and soluble S proteins [94–96], or for MERS-CoV [97].

**Fig. 5:**
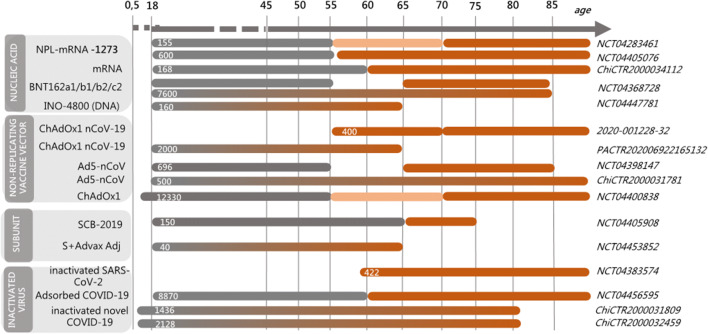
Ongoing clinical trials of COVID-19 vaccines specifically including the elderly population. Schematic representation of clinical studies specifically including older people in the selection criteria of volunteers. The platform used for each clinical trial is shown on the left. The identifier number of the clinical trial and the number of volunteers included (in brackets) are reported on the right. Bars represent the partition of volunteers according to the age range. Data are updated to 8th July 2020

The most advanced candidates for SARS-CoV-2 entered in human clinical testing with unprecedented rapidity employ nucleic acid (both mRNA and DNA), recombinant vaccine vectors (human or chimpanzee Adenovirus vectors), subunit S protein combined or not with different adjuvants, and inactivated SARS-CoV-2 virus. Other novel platforms based on the use of synthetic modified antigen presenting cells (APC) or cytotoxic T lymphocytes are also under study (Fig. 4). The platforms using mRNA, non-replicating viral vectors, and inactivated SARS-CoV-2 virus have already reached the clinical trial phase III. Some of the different platforms used may be tailored for specific population subtypes, such as the elderly, children, pregnant women, or immunocompromised patients [98]. In this regard, some of the ongoing clinical studies have specifically taken into consideration the older population, by including vaccination arms with people aged > 60 years. A schematic diagram of the ongoing phase I and II clinical trials that have included older adults is reported in Fig. 5. Enrolling older adult volunteers will help to better understand vaccination outcomes among the older population, who are most at risk of complications from COVID-19.

The ongoing clinical studies based on mRNA technology (mRNA-1273 from Moderna n. NCT04283461, and BNT162 from Biontech SE, n. NCT04368728) aim to evaluate the safety, tolerability, immunogenicity, and potential efficacy of different SARS-CoV-2 RNA vaccine candidates in the adult population, with a specific attention to older people (N.-N. Releases. NIH clinical trial of investigational vaccine for COVID-19 begins. 2020. https://www.nih.gov/news-events/news-releases/nih-clinical-trial-investigational-vaccine-covid-19-begins). The lipid nanoparticle-encapsulated mRNA-1273 vaccine, which encodes for the full-length S protein, is currently evaluated in a dose-ranging study in the adult population (18–55 years old), and in participants from 56 to 70 and > 71 years of age (Fig. 5). Similarly, the large dose-finding study with the BNT162 biological component (7600 estimated participants) based on the administration of mRNA coding for the full-length S protein, or for the two smaller receptor-binding domains, is going to test the immunogenicity in adults (18–55 years) and older adults (56–85 years).

An ongoing phase I/IIa trial (n. NCT04447781) is also aimed at evaluating the safety, tolerability, and immunological profile of the INO-4800 vaccine that, exploiting the DNA technology, contains a plasmid encoding the full-length S glycoprotein. The INO-4800 vaccine is administered by intradermal injection followed by electroporation in healthy adults aged 19 to 64 years.

Another platform that is currently specifically tested in older people is based on the Adenovirus type 5 vector that encodes the S protein from the SARS-CoV-2 strain (trials n. 2020-001228-32; PACTR202006922165132; NCT0439814; ChiCTR2000031781 and NCT04400838; Fig. 5). Different studies are ongoing, and one conducted in Canada is a dose-escalation designed study, from the younger adults (18 to < 55) to the older adults (65 to < 85). Another huge phase 2/3 study (n. NCT04400838) is aimed at determining the efficacy, safety, and immunogenicity of the candidate COVID-19 vaccine based on the chimpanzee adenovirus vector (ChAdOx1 nCoV-19) in healthy UK volunteers, specifically divided in adults (18–55 years old), elderly (over the age of 56), and children (5–12 years old). The ChAdOx1 platform has already been shown to be effective in the established rhesus macaque model of SARS-CoV-2 infection [99]. In this pre-clinical study, a single dose of ChAdOx1 nCoV-19 has protected six rhesus macaques from pneumonia caused by the virus [100]. Moreover, the ChAdOx1 has been used to develop investigational vaccines against several pathogens, including the closely related coronavirus responsible for the MERS [101]. Adenovirus-based vectors are characterized by a broad range of tissue tropism that covers both respiratory and gastrointestinal epithelium, the two main sites that express the ACE-2 receptor of SARS-CoV-2, even though a possible immunodominance mediated by vector genes rather than the transgenes should always be considered [102].

Using the traditional recombinant protein technology to express the spike protein, a trial sponsored by Clover Biopharmaceuticals AUS Pty Ltd. (n. NCT04405908) is assessing the safety, reactogenicity, and immunogenicity of multiple doses of SCB-2019 administered with AS03 adjuvant, or with CpG 1018 plus alum adjuvants. Data will be separately analyzed on adult (18 to 54 years of age) and elderly (55–75 years of age) healthy subjects enrolled in the study. In another study, the S protein has been administered with the Advax adjuvant (n. NCT04453852), a potent and safe immunopotentiator composed of delta inulin [103].

Four trials are testing in the elderly population the inactivated SARS-CoV-2 virus (n. NCT04456595; ChiCTR2000031809; ChiCTR2000032459), and one of these has been specifically performed only in people > 60 years (n NCT04383574; Fig. 5).

Numerous other vaccine developers have indicated plans to initiate human testing in 2020. Despite the several vaccine candidates (Fig. 4), challenges including the need for optimizing antigen design and adjuvant formulation define the number of doses needed, induce the optimal immune response without exacerbating the inflammatory and antibody-dependent response involved in possible lung disease, and fully define correlates of protection and duration of immune responses have to be considered [104].

Finally, a general consideration for the SARS-CoV-2 vaccine development regards safety issues that could arise with COVID-19 vaccines developed under the strong pressure of the pandemic situation. Animal studies on vaccines for SARS-CoV-1 and MERS-CoV report possible adverse effects mediated by vaccine-induced antibodies that have poor or no neutralizing activity [105]. Safety and efficacy are two indissoluble properties of a vaccine to be administered to billions of people globally and need to be accurately evaluated for every SARS-CoV-2 candidate.

## Systems biology and integrative analysis

The efforts in the development of COVID-19 vaccines can benefit from the availability of most advanced tools and high-throughput technologies to decipher the effective immune responses in the older population and the correlates of protection. Recent advances in systems biology integrating clinical, immunologic, and omics data can help to identify stable and robust markers of vaccine response and move towards a better understanding of SARS-CoV-2 vaccine responses in the elderly. Machine/statistical learning applied to multi-omics data from clinical studies promises to revolutionize vaccine development by illuminating the mechanistic drivers of protective immunity. The high-performance data acquisition methods in molecular and cellular biology push the field of bioinformatics for the development and use of tools that manage and integrate the different levels of biological complexity.

Application of the immunobiography approach could inform the stratification of elderly subjects and guide the implementation of vaccination strategies designed for specific elderly population clusters [87]. Mathematical modeling allows the combination of different networks involved in biological aging such as epigenetic networks, cell-cell networks, and population genetics and can allow to generate hypothesis on response to treatment or vaccination [106]. Recent progress in mathematical modeling can be utilized to generate biomarker models for prediction of disease and also response to vaccination taking into consideration biological age.

Currently, computational models have been applied to immunology data, for example, for the analysis of a high-dimensional dataset in vaccination studies [107, 108], but these models are limited to particular aspects [109, 110]. There is the potential for these models to become more sophisticated and to predict how responses to pathogens and vaccines are affected by pre-disposing factors [111, 112]. The systems vaccinology approach has been applied to characterize the immune response to different vaccines providing the proof-of-concept evidence of the capacity of systems approaches to delineate “molecular signatures” predictive of vaccine responses [113–131]. This approach has also been applied to identify molecular signatures induced by immunization with the rVSV-ZEBOV Ebola vaccine, recently approved for human use. Systems analysis has been conducted integrating clinical, immunologic, and omics data in clinical trials with different doses and in different continents (Vianello et al. 2020 submitted, Santoro et al. 2020 submitted).

Despite the great efforts made, unfortunately, many of the most useful clinical and multi-omics datasets are siloed in local databases to protect participant privacy and data confidentiality. Creation of secure, FAIR-compliant, federated learning databases in which predictive biological and mathematical models based on AI/machine/statistical learning can be developed, refined, and tested on distributed datasets would have an enormous impact in supporting a rational vaccine development.

## Concluding remarks

SARS-CoV-2 vaccines are urgently needed, and their design should take into consideration that the elderly are the main target population for vaccination. The pandemic is stimulating the research on vaccine development, and this should be a tremendous opportunity to specifically include age and gender as critical factors for vaccination approaches and effectiveness. While older adults are most likely to be severely affected by COVID-19, they also may be less responsive to vaccination. In the ongoing tremendous efforts for COVID-19 vaccine development, only a limited number of clinical trials have included the older fraction of the population in the study design, and the platforms used are not specifically designed considering the peculiarity of the elderly immune system. Indeed, vaccination strategies tailored for the SARS-CoV-2 infection in the elderly should take into consideration the delicate balance of immunosenescence and inflammaging with the immunopathological aspects of the SARS-CoV-2 infection, such as the cytokine storm reported in severe COVID-19. Therefore, the possible overlap between the factors hampering vaccination effectiveness in the elderly and those that boost the virulence and worsen the prognosis of SARS-CoV-2 infection should be carefully taken into consideration. Thus, vaccine formulations, such as adjuvants and vectors, should be specifically designed for stimulating the elderly immune system without exacerbating the inflammatory status. The ongoing efforts in COVID-19 vaccine development should fully exploit the availability of high-throughput technologies and recent advances in systems biology to decipher the effective immune responses in the older population and identify correlates of protection to guide towards SARS-CoV-2 vaccine strategies optimally designed to protect the older population.

## Data Availability

Not applicable
